# Aortic dissection type I in a weightlifter with hypertension: A case report

**DOI:** 10.1186/1757-1626-1-99

**Published:** 2008-08-18

**Authors:** Hossein Ahmadi, Shapour Shirani, Parin Yazdanifard

**Affiliations:** 1 Associate Professor of cardiovascular and thoracic surgery, Tehran Heart Center, North Kargar Street, Tehran, Iran

## Abstract

Acute aortic dissection can occur at the time of intense physical exertion in strength-trained athletes like weightlifters, bodybuilders, throwers, and wrestlers.

Rapid rise in blood pressure and history of hypertension are the most common causes of aortic dissection in athletes. It is a very tragic event because of its high mortality rate of about 32% in young patients. We report a case of aortic dissection in a young weightlifter with an extensive intimal tear of the aorta, from the sinus of Valsalva to the abdominal aorta.

## Introduction

Acute aortic dissection results from a tear in the intima and media of the aortic wall, with the subsequent creation of a false lumen in the outer half of the media and elongation of this channel by pulsatile blood flow. Dissection of the aorta is associated with a high degree of morbidity and mortality despite continuing improvements in diagnostic and surgical techniques [1], and hypertension is present as the most common cause in 70–90% of patients with aortic dissection [2]. A number of normal daily and athletic activities require isometric or static exercise. Sports such as weightlifting and other high-resistance activities are used by power athletes to gain strength and skeletal muscle bulk. These exercises significantly increase blood pressure, heart rate, myocardial contractility, and cardiac output. Hypertension has long been recognized as an important risk factor for the development of aortic aneurysms and dissections [1,3]. Also, it has been speculated that the very high blood pressure generated during the lifting of weights, particularly with staining accompanied by a Valsalva maneuver, may be the cause of an aortic intimal tear [3]. Pre-participation cardiovascular evaluation of young competitive athletes is warranted on the basis of the available evidence [4]. Patients with predisposing conditions to aortic dissection, including hypertension, should be sturdily encouraged to refrain from weightlifting. We present a case of aortic dissection in a young athlete with a history of hypertension.

## Case presentation

**Figure 1:**
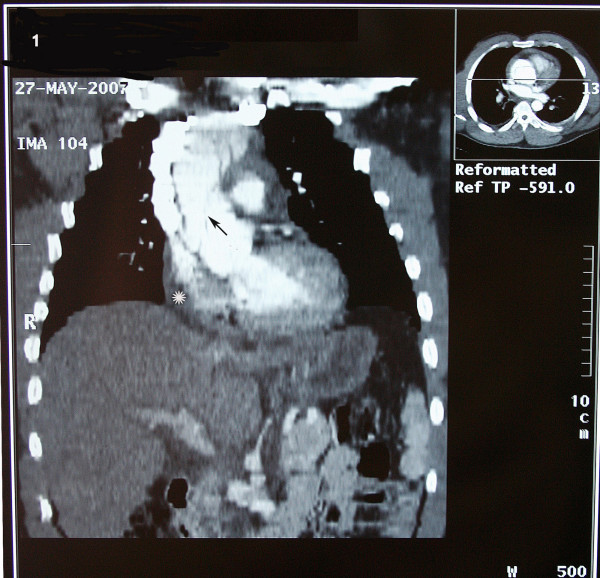
**Semicoronal reconstructed CT angiography reveals flap in the ascending aorta and arch (arrow).** The false and true lumens are patent. Hemopericardium is also seen (astrix).

**Figure 2:**
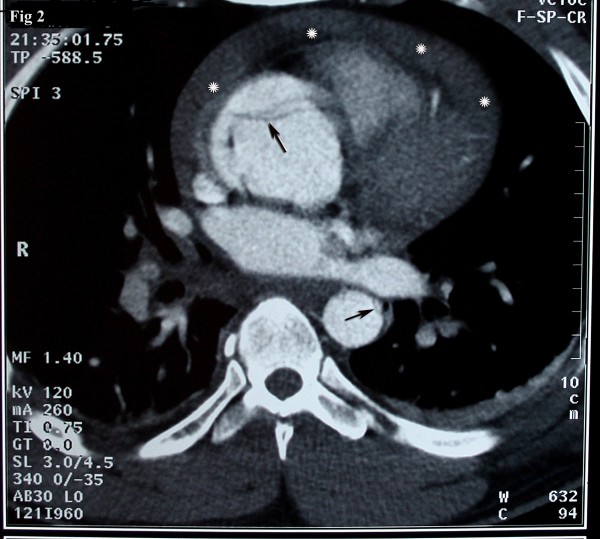
Axial source image of CT angiography reveals the flap in the ascending aorta compatible with type A of Stanford dissection: Arrows are showing the flap in ascending aorta and astrixes are showing hemopericardium.

**Figure 3:**
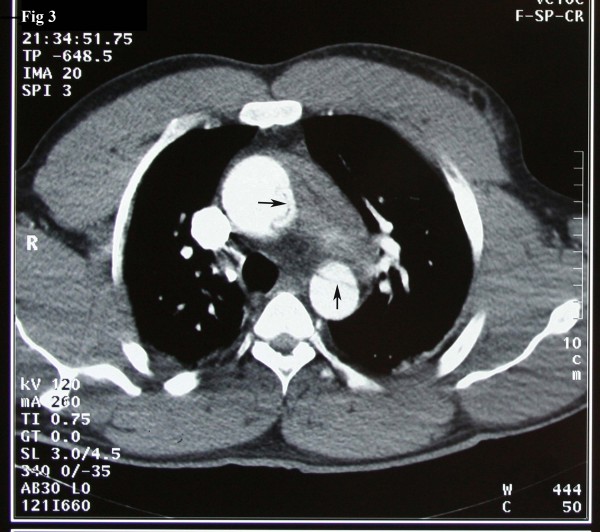
Axial source image of CT angiography reveals the flap in the descending aorta compatible with type A of Stanford dissection: Arrows are showing the flap in descending aorta.

**Figure 4:**
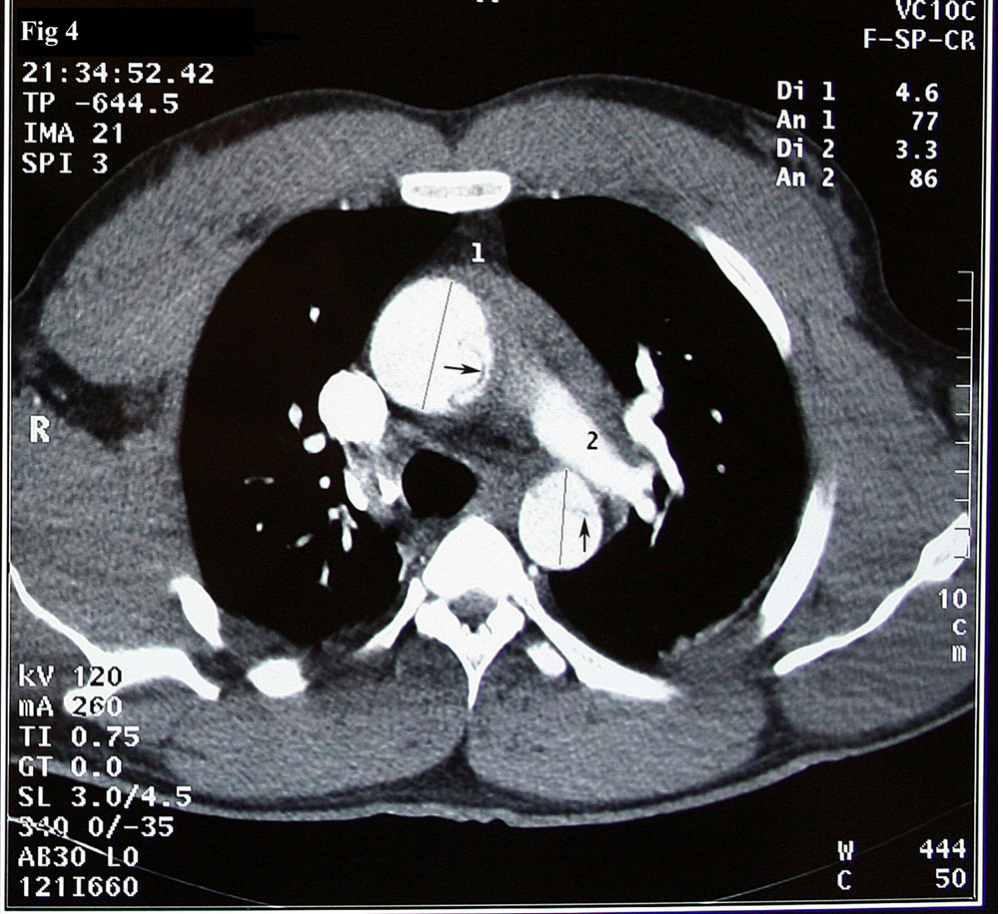
Axial source image of CT angiography show aneurysmal dilation of ascending aorta along with type A aortic dissection.

A 37-year-old Iranian man with a history of hypertension and long history of weightlifting was admitted to our hospital complaining of knifelike retrosternal chest pain, which was abrupt while lifting weight, accompanied by severe sweating and palpitation. Distal pulses were weakly palpable. Electrocardiography showed non-sinus arrhythmia, Wolf-Parkinson-White (WPW) syndrome, and Q in lead III and avf. Echocardiography findings were normal left ventricular size with concentric left ventricular hypertrophy (LVH), left ventricular ejection fraction (LVEF) of about 55%, dilated ascending aorta of about 56 mm, and mild mitral regurgitation. In addition, the intimal flap in the ascending aorta was seen collapsed. The diagnosis was obtained by computed tomography (CT) angiography, which showed a typical aspect of type A of Stanford classification of aortic dissection (ascending aorta, transverse arch, and descending thoracic aorta were involved) [fig 1]. The intimal tear was located just above the Valsalva sinus running to the abdominal aorta with hemopericardium [fig 2, 3]. Subsequently, the patient developed cardiac arrest and cardiopulmonary resuscitation (CPR) was performed. He was taken emergently for the surgical replacement of the aortic valve and repair of type I aortic dissection. Femoral artery cannulation, median sternotomy, and right atrial cannulation for total cardiopulmonary bypass (CPB) were carried out. At the opening of the pericardium, there was a typical ascending aneurysm which was extended to the origin of the innominate artery [fig 4]. After aortotomy, the entry site of the aortic dissection was identified anteriorly. The dissection was extended into the aortic arch and then into the aortic root. On the other hand, the ascending aorta was fully dissected with an extension of the proximal dissection toward the abdominal aorta. Because of the destruction of the sinus of Valsalva by the dissection, the Bental procedure was performed. The segment of the aorta containing the intimal tear was subsequently resected and replaced with a Dacron graft. Teflon felt was used and attached to the aortic wall with continuous sutures on the outer bound of it. Also, glue was applied to fill the entire space between the dissected fragile layers. CPB and aortic cross-clamp times were 300 and 240 minutes, respectively. After long-run anesthesia and cardiopulmonary bypass, the patient was weaned from CPB and admitted to intensive care unit (ICU) with inotropic support. Twenty-four hours after ICU admission, the patient developed a deep coma with pupils reactive to light, fully dilated left pupil, and no response to painful stimuli. Brain CT scan demonstrated acute infarction in the left cerebral hemisphere, right frontal lobe, brain swelling with midline shift, and subtentorial hernia. The blood pressure was dependent on inotropic support, and the cardiac rhythm was junctional. Cardiac arrest occurred after a few minutes. Unfortunately, CPR was not successful and the patient expired on the first day of operation.

## Discussion

The cardiovascular system adapts to exercise. Top-level training is often associated with morphological changes in the heart including increases in the left ventricular chamber size, wall thickness, and mass. The increase in the left ventricular mass as a result of training is called" athletes' heart" [5]. Morganroth and his colleagues [6] distinguished two different morphological forms of athletes' heart: a strength-trained heart and an endurance-trained heart. According to their theory, athletes involved in endurance training, sports with a high dynamic component like running, are presumed to demonstrate eccentric left ventricular hypertrophy, characterized an unchanged relationship between left ventricular wall thickness and left ventricular radius (i.e. ratio of wall thickness to radius), which means an increased left ventricular chamber size with a proportional increase in wall thickness. On the other hand, strength-trained athletes involved in mainly static or isometric exercise like weightlifting, bodybuilding, and wrestling, are presumed to demonstrate concentric left ventricular hypertrophy, which is characterized by an increased ratio of wall thickness to radius, which means an increased left ventricular wall thickness with an unchanged left ventricular chamber size. In addition to the aforementioned changes, in weightlifters as strength-trained athletes, cardiac output, heart rate, and blood pressure tend to increase. A rapid increase in the systemic arterial blood without a decrease in the peripheral vascular resistance, in combination with aortic medial degeneration, may contribute to the development of the aortic dissection [7]; this is an event that may occur in non-trained weightlifters or those with predisposing factors for aortic dissection, like hypertension, congenital cardiovascular disease (e.g. coarctation of aorta, congenital stenotic aortic valve, and unicuspid and bicuspid aortic valve), supravalvular aortic stenosis, connective tissue disorders (e.g. the Marfan syndrome and familial cystic medial degeneration syndromes), and fibromuscular dysplasia. Also in athletes who have mild-to-moderate aortic enlargement, an increased blood pressure due to heavy weightlifting, raises aortic wall stress to a level that begets aortic dissection [8]. Aortic dissection is a very tragic event because of its high mortality rate of about 32%, and the most common causes of death after aortic dissection involving the ascending aorta include the rupture into the pericardial cavity with resultant tamponade, occlusion of the coronary arteries, and free rupture into the chest or abdomen [2]. All athletes must be assessed for predisposing factors for aortic dissection, and all patients should be encouraged to undergo appropriate diagnostic studies like echocardiography and blood pressure monitoring while weightlifting to recognize possible predisposing factors for aortic dissection. Athletes who do have a problem should be encouraged to avoid or limit their exercise or activity by their cardiologist. It is vital that this disastrous event be prevented in young people.

## Conclusion

We strongly advise that athletes with one of the predisposing factors for aortic dissection eschew intense physical exertion. Unfortunately, survival after such dissections is extremely unanticipated because of the lengthy extension of the intimal tear, massive hemorrhage, and organ dysfunction.

## Abbreviations

 WPW: Wolf-Parkinson-White; CT: Computed tomography;  LVH: Left ventricular hypertrophy; LVEF: Left ventricular ejection fraction; ICU: Intensive care unit; CPR: Cardiopulmonary resuscitation; CPB: Cardiopulmonary bypass.

## Competing interests

The authors declare that they have no competing interests.

## Authors' contributions

AK carried out the surgery and was directly involved in the conception, design and drafting of the manuscript. SS participated in the diagnosis and treatment; also gave critical comments on the results. PY collaborated in the design of the study and was directly involved in drafting and revising the manuscript. All the authors read and approved the final manuscript.

## Consent section

Written informed consent was obtained from the patient's family for publication of this case report and accompanying images. A copy of the written consent is available for review by the Editor-in-Chief of this journal.
